# South African midwife specialists practice: Medico-legal experts’ experiences

**DOI:** 10.4102/hsag.v30i0.2851

**Published:** 2025-04-30

**Authors:** Kagiso P. Tukisi, Zelda Janse van Rensburg, Wanda Jacobs

**Affiliations:** 1 Department of Nursing Science, School of Health Care Sciences, Sefako Makgatho Health Sciences University, Pretoria, South Africa; 2 Department of Nursing Science, Faculty of Health Sciences, University of Johannesburg, Johannesburg, South Africa

**Keywords:** medico-legal experts, medical litigations, midwife specialists, South Africa, knowledge, skills, practice, utilisation

## Abstract

**Background:**

Medical litigations are rising and becoming a global challenge. The multilayered demands of midwifery-related conditions and the multiple responsibilities of the midwifery team contribute to possible litigations. Midwife specialists in South Africa are trained to provide specialised midwifery care to improve outcomes for patients with midwifery-related complications. While midwife specialists are equipped with specialised knowledge and skills, no specific practice regulations exist to protect them against possible medical litigations.

**Aim:**

The study aimed to explore and describe medico-legal experts’ experiences of midwife specialists optimally utilising their knowledge and skills.

**Setting:**

The context of the study is the public and private sectors of South Africa.

**Methods:**

A qualitative, descriptive and contextual research design was employed. Six medico-legal experts identified through a snowball sampling technique participated in virtual, semi-structured interviews. Data were analysed using Collaizi’s descriptive method.

**Results:**

Three themes with subthemes emerged. Results confirmed that medico-legal experts recognised the midwife specialists’ expanded knowledge and skill set. The medico-legal experts experienced a disconnect between training regulations and the current practice regulations. Midwife specialists’ practice was experienced as being guided by a moral obligation rather than prescribed regulations.

**Conclusion:**

Midwife specialists are yet to receive full legal recognition in South Africa, challenging midwife specialists’ assumption of autonomous and independent roles.

**Contribution:**

Midwife specialists face potential risk of involvement in medical litigations under the current legislative framework. The findings of this study may guide the formulation of a scope of practice (SOP) that legally guides midwife specialists’ practice in South Africa.

## Introduction

Midwife specialist (MS), in the South African context, refers to a professional nurse and midwife with advanced expertise, knowledge and skills in midwifery, according to the South African Nursing Council (SANC 1993). Midwife specialists undergo post-basic midwifery and neonatal nursing training in National Qualification Framework (NQF) level 6 and postgraduate midwifery NQF 8, leading to their heightened clinical acumen, knowledge and skills (SANC 2020). The MS training and specialists programme was first introduced in South Africa to respond to address the increase of maternal and neonatal mortalities and morbidities in the country (Maree, Yazbek & Leech 2021). The increase in maternal and neonatal mortalities was linked to the shortage of doctors in clinical facilities (Maree et al. 2021; Zihindula et al. 2019). A resolution was taken to enrol registered midwives in a postgraduate midwifery programme to expand their knowledge and skills to enable them to act in the case of high-risk maternal and neonatal conditions and complications autonomously and independently (Maree et al. 2021; Okoroafor & Chistmals 2023). South Africa followed the trends of countries such as Liberia and Uganda, where there are similar circumstances of critical shortages of doctors in rural parts of the country, which makes handling critical patients challenging (Dolo et al. 2016; Kumakech et al. 2020).

Midwife specialists, at the end of their training, are equipped with advanced knowledge and skills (Tukisi et al. 2024; Williams et al. 2019). According to the literature on primary healthcare reengineering, MSs are responsible for the midwifery management of patients during the antenatal, intrapartum, postnatal and neonatal periods (Basu 2023; Li et al. 2020). However, the literature reveals that MSs often practise their profession with some limitations (Williams et al. 2019; Tukisi et al. 2024). This limited practice of MSs was linked to the unclear roles of MSs in clinical practice (Tukisi et al. 2024). The existing literature also points out that the SANC has yet to define the specific MS roles and responsibilities in clinical practice through a scope of practice (SOP) specifically for MSs (Toll et al. 2023; Tukisi et al. 2024)

The current scope of practice, Regulation 2127 (South African Government 2022), specifically guides the practice of midwives with specific reference to the roles and responsibilities of basic midwives. South African Nursing Council on the other hand has published the ‘competencies for midwife specialist’ (SANC 2020) that refer to an MS as a practitioner with advanced knowledge and skills that must be utilised to respond to complicated maternal, neonatal and reproductive health needs.

The SANC serves as an Education and Training Quality Assurance body (ETQA) and regulates MS education, training and clinical practice according to the *Nursing Act 33 of 2005* (SANC 2005). Midwife specialists, as healthcare practitioners, are responsible for their professional acts and responsibilities (Buchanan et al. 2022). Consequently, the MS practice is guided by regulations from the SANC as a professional regulatory body (SANC 2020). The current legal restrictions surrounding the MS practice put the MSs at a potential risk of medical negligence (Kirkham 2020). Medical negligence refers to the injury the patient suffers secondary to the direct actions or inactions of the health practitioner or in the instances of health practitioners’ deviation from the standard medical procedure (Miziara & Miziara 2022).

Although the aim of the MS training programme in South Africa was to improve maternal and neonatal morbidity and mortality (Sellers, Dippenaar & Da Serra 2018), the medical litigations involving MSs and obstetric teams in the country are rising (Mokoena 2021). Currently, MSs practise in a litigation-prone environment as evidenced by a dramatic rise in litigation costs since 2014, with an estimated annual payout of six and a half billion rand (Pitt 2022). Midwife specialists are at an even higher risk of involvement in litigations because of their involvement in the management of obstetrical complications (Eck 2021). Cerebral palsy (CP), a group of disabilities secondary to brain damage because of intrauterine foetal hypoxia and traumatic labour, accounts for 50% of the recorded medical litigations (Pitt 2022; Prinsen 2022; Sellers et al. 2018).

### Aim

The study aimed to explore and describe medico-legal experts (MLEs) experiences in MS’ optimal utilisation of their knowledge and skills in the public and private health facilities in South Africa. Although the MLE is not directly involved in MS practice, MLEs are familiar with the knowledge and skills of the MSs based on their direct legal involvement in MS practice. Medical-legal expert serves as a representative who scrutinises and investigates medical practice when cases of negligence and medical litigations are reported. The MLEs’ experiences may shed light on the hesitance of MSs to optimally utilise their knowledge and skills in practice.

## Research methods and design

### Study setting

The context of the study is the public and private sectors of South Africa. The public health sector is a government-funded health service that caters accessible and affordable healthcare services to 71% of South Africans. Antenatal healthcare services are accessible from primary healthcare clinics, community health centres and midwife-led obstetric units. The midwifery team, comprising midwives and midwife specialists, renders antenatal, intrapartum and postnatal services. In addition, hospitals cater to high-risk patients who require the midwife specialist’s care. The private hospital groups, on the other hand, cater to 29% of the total South African population. The MLEs in the study held experience in litigations involving MSs from both health sectors across South Africa. At the time of data collection, MLEs from four of the nine South African provinces (Gauteng, KwaZulu-Natal, Free State and Limpopo) were represented. Recruitment and participation of the MLEs were found to be challenging because of their demanding professional commitments.

### Design

The research design was qualitative, exploratory and descriptive in nature, with a phenomenological approach to understand the experiences of the MLEs regarding utilisation of MS knowledge and skills. The MLEs experience the MSs’ involvement in medical litigations firsthand and were therefore viewed as knowledgeable about the phenomenon under study (Polit & Beck 2020).

### Population and sampling

The population comprises six MLEs, comprising of both MSs who had experience in medical litigations or worked as medical-legal consultants, and lawyers and attorneys who deal with medical litigations. A non-probability, purposive sampling strategy was employed. The sampling strategy allowed the researcher to hand-pick participants who specialise in medical litigations that involve MSs and who were willing to share their experiences regarding optimal utilisation of MS knowledge and skills. After that, a snowball sampling strategy was followed to identify participants who further met the inclusive criteria. The participants needed to be either lawyers or attorneys who were registered as such under the Legal Practice Council of South Africa and who were experienced in medical-legal litigations involving the MSs. In addition, the MS recruited needed to be registered with SANC as midwives and MS. Furthermore MS needed to experienced as consultants for lawyers or attorneys, reviewing medico-legal cases.

The researcher collected data from MLEs representing four out of nine provinces in South Africa.

### Data collection

The researcher arranged appointments with the MLEs’ assistants to share the research information and consent forms. The MLEs’ assistants then scheduled an appointment for the virtual interview via Microsoft teams^TM^ at a convenient date and time for the participant and the researcher. Consent was obtained to record the virtual interviews on Microsoft Teams beforehand. Each interview lasted between 60 and 90 min.

The participants were asked to deliberate on one central question, which was: *As a medical-legal expert, what is your experience in MS optimally utilising their knowledge and skills?*

Probing and other interview techniques were used to encourage the depth and richness of the data obtained. The first two interviews were regarded as a pilot study to rephrase the research question in case it needed to be explained to the participants (Polit & Beck 2020). There was no need to rephrase the research question, and the participants from the pilot study formed part of the primary sample. After the transcription of the sixth interview, it was determined that the same themes emerged, indicating data saturation.

### Data analysis

The seven steps of Collaizi’s descriptive phenomenological method were followed to analyse the data (Polit & Beck 2020). The researcher had no direct or indirect relationship with any participants other than being an MS (*accoucheur*) himself.

All the interviews were conducted on Microsoft Teams^TM^ and were subjected to audio recordings consented to by the participants. Although Microsoft Teams^TM^ has an auto-transcription function, the researcher verified the transcriptions against the audio recordings to ensure the accuracy of the transcriptions (LoBiondo-Wood & Haber 2021). The researcher also asked the participants to leave their cameras on to observe non-verbal cues as field notes. Moments of silence were also noted to probe the participants to elaborate on their responses.

The researcher read the verbatim transcriptions to submerge themself in data to gain a deeper understanding of the participant’s lived experiences. The significant statements were extracted from the whole data and clustered into themes and subthemes that detailed the participants’ lived experiences of MS’ optimal utilisation of their knowledge and skills. Copies of the transcribed interviews were sent to an independent coder who also analysed the data. A consensus discussion was held between the researcher, independent coder and supervisors to confirm the themes and subthemes after the findings were contextualised using literature.

### Trustworthiness

To increase the trustworthiness of the study, the five principles of Lincoln and Guba (1986) (credibility, transferability, dependability, confirmability and authenticity) were adhered to (Adler 2022). Credibility was ensured by keeping an audit trail of all the information about the study and prolonged engagement with the participants to establish rapport and trust. The researcher preliminarily analysed data and employed an independent coder to confirm the emerging themes and subthemes. Member checking was done following the data analysis and the preliminary findings were shared with the participants to validate the results. Adequacy of the references was ensured by recording the interviews on Microsoft Teams^TM^. Dependability was achieved by a code-recode data analysis method. This means that the data were coded over an extended period to ensure consistency. The independent coder also assisted in co-coding the data independently from the researcher. The themes of the analysed data were finalised during a consensus meeting between the researcher and the independent coder (Stahl & King 2020). To ensure the transferability and generalisability of the findings, the researcher provided demographic information of the participants, which justified their selection and relevance for participation in the study (Adler 2022). A clear description of the research methodology was also given. Conformability was ensured by transcribing the interviews verbatim and documenting direct participant quotations. The researcher kept an audit trail of all the study documentation, including audio recordings, written notes and verbatim transcriptions (Stahl & King 2020). To ensure the authenticity of the study, the researcher captured the participant’s experiences and emotions to increase understanding of the context (Stahl & King 2020).

### Ethical considerations

Ethical approval to conduct the study was obtained from the University of Johannesburg Research Ethics Committee and Higher Degrees Committee (REC-1279-2021; HDC-01-154-2021). The participants are experts in their respective fields and are not considered vulnerable. Consequently, they were approached and invited to participate in the study. All participants provided informed consent for their involvement. The researcher emphasised that participation is voluntary, and each participant has the right to withdraw from the study at any time. Each participant was assigned a code for data discussion to protect their anonymity. Data were stored on a password-encrypted computer to ensure confidentiality.

## Results

Data were collected from six MLEs (participants) who shared their MS experiences utilising their knowledge and skills. The participants were collectively called MLEs based on their knowledge and experience of MS medical litigations.

### Participants’ demographic characteristics

The MLEs comprise three qualified lawyers with bachelor’s and master’s degrees in law. Their years of experience in law ranged between 11 and 16 years at the time of data collection. The MLE sample further included three MSs with 10 to 17 years of experience in midwifery, including consultation work for lawyers on medical litigations. One MS held a diploma in nursing and midwifery; two other MSs held a bachelor’s degree in nursing and midwifery. All three MSs held a master’s degree, and one had a doctoral degree in Midwifery and Neonatal Nursing. The participants’ characteristics are summarised in Table 1.

**TABLE 1:** Depiction of participants’ socio-demographic information.

Participant code	Qualifications	Years of experience	Sector	Province
MLE 1	Bachelor of law Master of law	11	Public and Private	Gauteng
MLE 2	Bachelor of law Master of law	14	Public and Private	Gauteng
MLE 3	Bachelor of law Master of law	16	Public and Private	Limpopo
MLE 4	Bachelor of nursing and midwifery (R425) Master of Neonatal Nursing (R.212) Doctor Curations: Maternal and Child Nursing Science: Advanced midwifery and Neonatal Nursing Science	17	Public and Private	Gauteng
MLE 5	Bachelor’s in nursing and midwifery (R425) Diploma in post-basic midwifery (R.212)	10	Public and Private	Free State
MLE 6	Diploma in nursing and midwifery (R425) Diploma in post-basic midwifery (R.212) Master’s degree in Midwifery and Neonatal Nursing	15	Public and Private	KwaZulu-Natal

MLE, medical-legal expert; R, Regulation number.

### Presentation of the findings

The following three themes emerged from the data analysis, as indicated in Table 2. Misalignment of education and practice regulations, unlawful midwife specialists’ professional practice and factors exposing midwife specialists to litigation. Table 2 also summarises the study’s results according to themes and subthemes.

**TABLE 2:** Summary of themes and subthemes.

Themes	Subtheme
1 Misalignment of education and practice regulations	1.1 Legal approval of education and training guidelines 1.2 Absence of specific practice regulations
2 Unlawful midwife specialists’ professional practice	2.1 Practice out of moral obligation 2.2 Disregard the existing scope of practice
3 Factors exposing midwife specialists to litigation	3.1 Ambiguity of the current scope of practice 3.2 Midwife specialists’ acts and omissions 3.3 Unlawful task shifting 3.4 Knowledge and skills decay

### Theme 1: Misalignment of education and practice regulations

Under this theme, it became evident that the education and training of MSs are approved and recognised by the SANC. According to the MLEs, a curriculum, competencies and training regulation guides MS training in nursing education institutions. However, the specific practice regulations to guide MSs’ professional acts in clinical facilities are yet to be developed.

#### Subtheme 1.1: Legal approval of education and training guidelines

The MLEs highlighted that the education and training regulations are explicit about the knowledge and skills to be embedded in the MS during training. One participant explained:

‘It is known that they have expanded knowledge and evidence exists on the training regulations.’ (MLE 2)

The MLEs explained that the published competencies for midwives’ specialists guide the knowledge and skills to be mastered by the MS. To ensure that MSs’ acquire such knowledge and skills, the training guidelines for MSs are followed. One participant explained:

‘There is a document on competencies of advanced midwives which set out what the advanced midwife is supposed to be like in terms of responsibilities’. The training regulations is R.212 [*Training regulation midwives leading to their registration as midwife specialists*], that’s the regulation they [*MS*] train under.’ (MLE 4)

#### Subtheme 1.2: Absence of specific practice regulations

The MLEs explained that the current SOP is not aligned with the specialist’s knowledge and skills in all clinical facilities. The MLEs argue that this might be an omission as the MSs were supposed to be conversant with the regulations relevant to their practice upon completion of studies. One MLE elaborated:

‘So, at this point we know that everything that the midwife specialists have been trained on is not linked to their scope of practice. Which is something that was not supposed to be the case, because a specialist was supposed to exit the college knowing all advanced roles and responsibilities awaiting him/her in the clinical setting.’ (MLE 5)

One participant pointed out that it is clear from the training regulations that the MSs are prepared with expanded knowledge and skills. However, the current SOP limits the specialist’s practice. The participant mentioned:

‘We have the midwife specialists and neonatal nursing specialists, highly specialised, but their scope of practice is very limited … the legal documents that are required to set out exactly what an advanced midwife can do doesn’t support that.’ (MLE 1)

The participants also highlighted that the current practice regulation excludes the practice of MSs and only details the basic midwives’ roles and responsibilities. One participant elaborated:

‘The current regulations are specific to basic midwives practice and do not apply to a midwife specialist. We have conditions for midwives practice and the scope of practice of the registered nurse and midwives.’ (MLE 6)

### Theme 2: Unlawful midwife specialists’ professional practice

According to the participants, MSs’ acted out of duty and obligations while executing their midwifery responsibilities. Acting from moral obligation sometimes led to disregarding the current SOP, potentially landing MSs into trouble with the regulatory body.

#### Subtheme 2.1: Practice out of moral obligation

The participants argued that the absence of specific practice regulations renders the MSs’ practice somewhat illegal. Two of the participants mentioned:

‘Because there is no scope of practice for them then we can’t even legally say you are an advanced midwife.’ (MLE 1)

‘A lot of advanced skills which are not linked to the current scope of practice. So, it appears specialists know more than they should know.’ (MLE 3)

Although the MSs’ optimal practice is restricted, the MLEs expressed that the MSs sometimes apply their knowledge and skills to benefit the patients. The participants argued that the midwifery profession has a deep sense of moral obligations. One participant elaborated:

‘Midwife specialists deal with complicated patients daily and they run a risk of losing a patient. So, in those instances, they [*MS*] feel obliged to intervene and save the patient.’ (MLE 4)

#### Subtheme 2.2: Disregard the existing scope of practice

The participants highlighted that the MS moral-driven interventions attempting to respond to midwifery or neonatal complications are likely to contravene the current SOP. One participant expressed:

‘Attempting to save the patient is one thing, but that still doesn’t take away the fact that in your [*MS*] actions to save the patient, you [*MS*] were not within your scope of practice.’ (MLE 5)

The participants explained that MSs may inevitably act outside of the SOP because of their work circumstances. The critical shortages of doctors in clinical facilities give rise to instances where MSs have to manage patients unaided. The participants mentioned:

‘The shortage of staff is a major issue, and I am not only referring to the midwives, but I am referring to also the doctors. You find that the doctor is not available at that time and the patients must be attended. That’s how we end up with the issue of operating outside the scope of practice, it is because there is no doctor.’ (MLE 4)

### Theme 3: Factors exposing midwife specialists to litigation

The participants experienced that the MSs were at risk of being implicated in litigations because of many factors. All the factors were associated with the current SOP, which made it difficult for MSs to be clear of their roles and responsibilities and accept unauthorised clinical orders. In addition, MSs are in constant fear of litigation, which can cause them to refrain from interventions that could potentially result in litigation.

#### Subtheme 3.1: Ambiguity of the current scope of practice

The MLEs pointed out that the current SOP is ambiguous and, therefore, it is challenging to exercise their roles and responsibilities during clinical situations according to guidelines of care. One participant mentioned:

‘The scope of practice is vague, it is not specific, it is not clear in terms of what you can do and cannot do. When you could sit down, you will realise that there are limitations with the scope of practice.’ (MLE 5)

The participants cautioned that the ambiguity of the SOP makes its contents subject to interpretation, exposing MSs to litigations, as anyone can have a different interpretation.

One participant mentioned:

‘It [*SOP*] is vague and when you read through it boils down to interpretation point of view and often, it is the reason why they are charged.’ (MLE 3)

#### Subtheme 3.2: Midwife specialists’ acts and omissions

The ambiguity of the SOP makes it challenging for MSs to apply their knowledge and skills. Therefore, MSs are often in a quandary whether to act or omit some of the clinical interventions as the SOP is unclear on the roles and responsibilities of MSs. The participants pointed out that both acts and omissions may potentially lead to litigations: It was mentioned that:

‘Regulation on acts and omissions makes it difficult for the midwife specialists to practice especially with the scope of practice that is not clear. One can be charged.’ (MLE 2)

The participants stressed that MSs are vulnerable to litigations when they behave proactively in a clinical situation where advanced clinical interventions are required. One participant mentioned:

‘So, the advanced [*Midwife specialist*] as a person who has advanced knowledge and skills as I have explained earlier is in a tight spot here. Negligence in the context of the midwife and childbirth could refer to what you shouldn’t have done but you did.’ (MLE 5)

The participants stressed that such actions could be a direct contravention of the SOP which could lead to MSs facing medical litigations especially in cases where the interventions had undesirable health outcomes. The participants shared:

‘I am saying this because for advanced midwives as soon as something goes wrong and you are requested to respond to such an allegation, no explanation whatsoever is going to be meaningful. It stops at: You acted outside your scope of practice.’ (MLE 1)

‘The focus is on legality of your practice. No one is concerned about that the fact of the matter is you worked outside what is prescribed for you! You have overstepped your mandate!.’ (MLE 6)

The participants highlighted that the MS’ choice to refrain from the application of their knowledge and skills opens opportunities for possible implications in litigations. The MSs may be charged for omitting life-saving interventions. One participant explained:

‘Negligence in the context of the midwife and childbirth could refer to what you could have done, but you didn’t do or to what you.’ (MLE 5)

The presence of the ‘competencies for midwife specialists’ provided by SANC, raised awareness regarding MS knowledge and skills in place. The ambiguity of the SOP may cause the interpretation that the MSs deliberately withheld a life-saving intervention, especially in cases of deleterious outcomes for a patient when a physician was unavailable. One participant shared:

‘It is known that they [*Midwife specialists*] have expanded knowledge and evidence exists on the training regulations,o sometimes leaving out some of the interventions for the doctor can be read as omission, so in the case of midwife specialist it can still be argued that in the absence of a doctor you are supposed to intervene because it is what you are trained to do.’ (MLE 2)

An MS’s choice not to intervene may be interpreted as withholding necessary care from patients, and litigation may be filed for that reason. In addition, the awareness of MS knowledge and skills could be used to aggravate an even harsher punishment on the MS. The participants mentioned:

‘The fact that one has an additional qualification act against midwife specialists because it is expected of them to be extra vigilant and know more than a basic practitioner so one can get a harsher sentence because it will be raised during aggravation.’ (MLE 1)

‘That is also another opportunity for them to be charged because if you omit then they can still go back to your training regulations and pinpoint all the things that they have studied and can prove that they have that knowledge and skill.’ (MLE 5)

#### Subtheme 3.3: Unlawful task shifting

Another factor that could potentially lead the MSs to litigation is task shifting from obstetricians’ (OBS) responsibilities to MS. The OBS’s awareness of the MS knowledge and skill set led to the OBS delegating some of the responsibilities to MS, oblivious of the limitations within the current SOP for the MS. The participants shared:

‘Doctors are aware of different levels of knowledge and skills set between the midwife and the advanced midwife. But they may be unaware that there is an issue of legal protection.’ (MLE 1)

‘Verbal communication can take place telephonically and in person, the midwives do contact the obstetricians or paediatricians should they pre-empt that there is a problem coming during birth or there is a compromised baby. Based on the urgency, the doctor may order some interventions.’ (MLE 4)

Implementation of orders to perform invasive clinical interventions on the patients from the physicians increases the MSs’ vulnerability to litigations. Although the MSs are knowledgeable and skilled, they might not be legally permitted to practice according to doctors’ expectations. Therefore, the possibility of involvement in litigations increases. One participant mentioned:

‘So, I will say the midwife is vulnerable to the charges because, say, for instance, the same intervention telephonically ordered by the doctor went wrong, the baby did not make it! Then, with reference to the scope of practice, the midwife specialist can still be held accountable for performing an act she is not legally permitted to do.’ (MLE 6)

#### Subtheme 3.4: Knowledge and skills decay

The generalised fear of litigations among the MSs made them weary of professional acts that could lead to litigations. Consequently, MSs avoided performing some interventions at the risk of negligence. One participant shared:

‘At this point, it looks like everything the advanced midwife will do is wrong because there are no specifics in the scope of practice. So, they [*MS*] avoid some of the things.’ (MLE 1)

The MSs’ avoidance of some interventions was evidenced by the clinical records, which show a decline in the productivity of MS. One participant explained:

‘We are noting a decline in their [*MS*] productivity. At this point, it seems like the maternity wards are now filled with people who just don’t want to be there anymore. The records do not detail the actions expected from the midwife specialists.’ (MLE 3)

The participants argued that a lack of practice contributes to the potential loss of specialised knowledge and skills. With the marked loss of MS knowledge and skills, the participants stressed that the MSs might not be able to respond to the patients’ problems, which is yet another reason for being implicated in medical litigations. The participants shared:

‘If you are not practicing, then you are slowly going out of practice; you are likely to struggle with managing a complication and you can easily lose a patient. Just like that you are in for a litigation, this is because a husband expected his wife to come out of the hospital alive with a newborn baby only to be told that the mother died, or both the mother and baby had died.’ (MLE 2)

‘I have seen it multiple times with my own eyes that they do not know how a neo-puff works, for a bag-valve resuscitation. They don’t understand it. They don’t understand how to assemble the neo-puff, to set the inspiratory pressure, the expiratory pressures. They don’t know to do the settings, uhm! The neo- puff which forms part of the emergency preparedness for that mother and the baby, the resuscitation is still delayed and that leads to litigations.’ (MLE 4)

## Discussion

The study sought to explore and describe the MLEs’ experiences of litigations involving MSs in the public and private sectors of South Africa. The MLEs experienced that although MSs are knowledgeable and skilled, their practices have limitations. The MSs’ limited practice could stem from the evident misalignment between the education, training and practice regulations. The misalignment between the education and practice of nurses and midwives’ specialists is a global challenge in nursing and midwifery (Mayra, Padmadas & Matthews 2021). According to the literature, the education regulations for specialist training in Uganda and Liberia allowed for the expansion of knowledge, whereas the practice regulations remained unchanged (Herndon & Vanderlaan 2024; Mayra et al. 2021).

According to the MLEs, the MSs’ practice requires a specific SOP that appreciates the specialist’s knowledge and skills. An SOP provides a set of parameters within which a nurse or midwife may practice as well as the law-specific restrictions that determine the tasks nurses and midwives may undertake while caring for patients (Downie et al. 2023). Currently, the MS practice raises legal questions; consequently, the MSs may not assume autonomous roles (Veenema et al. 2021). In addition, the current SOP serves as a framework for hospital policies guiding the MS practice, further restricting the MSs’ autonomous functions (Boman, Levy & Fagerström 2020). Consequently, the MSs were hindered from exercising their specialists’ discretionary powers to manage patients in clinical facilities.

Midwife specialists are primary caregivers in clinical facilities and attend to complicated conditions and life-threatening obstetrical emergencies (Wu, Lopez & Nichols 2022). Literature highlights that the onset of obstetrical emergencies prompts the MS’s immediate responses to improve the patient’s outcomes (Mashamba & Ramavhoya 2021; Raoust et al. 2022). The MS’s reactions to the patients in need directly apply the principle of beneficence and non-maleficence because the MS’s concern is the patient’s well-being (Wu et al. 2022).

Unfortunately, the MS practice in obstetrical emergencies may override the parameters set out in the SOP, potentially resulting in litigation. Consequently, MS was sanctioned regarding SANC regulation on acts and omissions (SANC 2014). The actions of midwives outside the SOP may contribute to the high number of litigations involving the midwives and MSs. In 2019, there were three cases of nurses, midwives and MSs who acted outside of the SOP (SANC 2021). According to the statistics from the SANC, midwifery-related cases accounted for 30% of cases that were heard in 2021 which include midwives and MSs (SANC 2014, 2021).

Unfortunately, the MS practice is in a punitive context and is litigation-prone because of various factors, according to the MLEs. The MLEs interpreted the current SOP as vague and ambiguous, making interpretation inconsistent. The ambiguity of SOP makes it challenging for nurses, midwives and MSs to be aware of their specific professional roles and responsibilities, consequently making it difficult for the professional practice to be consistent and standardised (Feringa, De Swardt & Havenga 2018; Kruger & McCann 2018). Ideally, the SOP should clearly describe MSs’ exact roles and responsibilities within a clinical context to avoid unintentional breaches of SOP (Koch 2023).

According to the MLEs, the absence of the SOP breeds an opportunity for unlawful task shifting from the doctors to the MS. The task shifting is brought about by the blurred professional boundaries between the doctors and MSs, as the role of the MS is unclear (Adeyemo, Morelli & Kennedy 2022). Task shifting is likely to lead to MS contravention of the practice regulations. Many midwifery and obstetric interventions are invasive, whereas SANC cautions against performing invasive procedures (Keshri & Garg 2021; SANC 2014).

The study found that the fear of litigations and the intentional omission of interventions have led to a gradual loss of knowledge and skills among the MS, another source of litigations. The fear of litigation was caused by the absence of practice regulations such as SOP and the job description of the MSs (Kirkham 2020; Mokoena 2021; World Health Organization [WHO] 2019). The gradual loss of knowledge and skills suggests that the MSs were not always prepared to intervene during complications leading to adverse events, which is the main reason for MSs’ involvement in litigations.

## Limitations and recommendations

Access to the participants was challenging because of the scarcity of professionals specialising in medical litigations in midwifery. The access was further limited by MLEs’ demanding work schedules, which resulted in a small sample size.

The findings of this study have shown the misalignment of education and practice regulations and the associated complexities within the clinical practice. The study findings suggest a need for collaboration between the SANC and the public and private health sector to review the current practice regulation. These findings could influence midwifery education as the MS’ education needs to be adjusted to allow training to be linked to what is legally permissible for application in clinical practice. In addition, the review of the current practice regulation may eliminate the fear of medical litigations, thus allowing for MSs’ optimal utilisation of their knowledge and skills.

## Conclusion

The findings in this study identified gaps between the education, training and practice regulations of MS. The findings also revealed various factors that may predispose the MS to medical litigations. A qualitative and descriptive research design provided a complete description of the MLE’s experiences of MS’s utilisation of their knowledge and skills. The MLEs in the study acknowledged the specialised knowledge and skills of the MS, demonstrating the recognition of midwifery education in South Africa. However, this study revealed that the current SOP needs to be aligned with MS’s expanded specialised knowledge and skill set. The ambiguity of the current SOP may be misinterpreted by midwives and MS, potentially leading to medical litigations. With the prevalence of medical litigations on the rise, MSs find themselves weary of specialised practice, which unfortunately leads to dormancy and gradual loss of knowledge and skills.
